# Does vasopressor therapy have an indication in hemorrhagic shock?

**DOI:** 10.1186/2110-5820-3-13

**Published:** 2013-05-22

**Authors:** François Beloncle, Ferhat Meziani, Nicolas Lerolle, Peter Radermacher, Pierre Asfar

**Affiliations:** 1 Laboratoire HIFIH, UPRES EA 3859, IFR 132, Université d’Angers, PRES LUNAM, Angers, France; 2 Département de réanimation médicale et de médecine hyperbare, CHU d’Angers, 4 rue Larrey, Cedex 9, F-49933, Angers, France; 3 Laboratoire de Biophotonique et Pharmacologie, UMR 7213 CNRS, Université de Strasbourg, Strasbourg, France; 4 Service de réanimation médicale, Nouvel Hôpital Civil, Hôpitaux universitaires de Strasbourg, 1, place de l'hôpital, 67031, Strasbourg, France; 5 Sektion Anästhesiologische Pathophysiologie und Verfahrensentwicklung, Klinik für Anästhesiologie, Ulm, Germany

**Keywords:** Hemorrhage, Shock, Trauma, Critical care, Vasopressors, Experimental studies, Clinical studies

## Abstract

This review aimed to answer whether the vasopressors are useful at the early phase of hemorrhagic shock. Data were taken from published experimental studies and clinical trials. Published case reports were discarded. A search of electronic database PubMed was conducted using keywords of hemorrhagic shock, vasopressors, vasoconstrictors, norepinephrine, epinephrine, vasopressin. The redundant papers were not included. We identified 15 experimental studies that compared hemorrhagic shock resuscitated with or without vasopressors, three retrospective clinical studies, and one controlled trial. The experimental and clinical studies are discussed in the clinical context, and their strengths as well as limitations are highlighted. There is a strong rationale for a vasopressor support in severe hemorrhagic shock. However, this should be tempered by the risk of excessive vasoconstriction during such hypovolemic state. The experimental models must be analyzed within their own limits and cannot be directly translated into clinical practice. In addition, because of many biases, the results of clinical trials are debatable. Therefore, based on current information, further clinical trials comparing early vasopressor support plus fluid resuscitation versus fluid resuscitation alone are warranted.

## Review

## Introduction

The use of vasopressors, such as norepinephrine for the hemodynamic management of hemorrhagic shock, may be considered in the early phase of resuscitation and is a common practice among several prehospital and hospital emergency teams in Europe except in the United Kingdom [1]. Nevertheless, it is worth noting that there is no mention of vasopressor use in North American textbooks and European recommendations [2,3]. Thus, it seems legitimate to question the appropriateness of its use as well as the choice of the drug to be used. In this article, we will first attempt to understand whether there are physiopathological arguments justifying vasopressor use during hemorrhagic shock. Next, our purpose is to scrutinize data stemming from animal experiments. Lastly, we will analyze the few human studies on this subject published thus far. Be aware that the goal of our investigation is not to debate about the hemodynamic endpoints notably in terms of blood pressure to be achieved during hemorrhagic shock resuscitation according to the different phases, namely the prehospital phase, the phase preceding hemorrhage control, and lastly the phase following hemorrhage control.

## Methods

### Data source

Published experimental studies and clinical trials. Published case reports were discarded.

### Data extraction

A search of the electronic database PubMed was conducted using the keywords of hemorrhagic shock, vasopressors, vasoconstrictors, norepinephrine, epinephrine, and vasopressin. The redundant papers were not included.

### Data synthesis

We identified 15 experimental studies that compared hemorrhagic shock resuscitated with or without vasopressors, three retrospective clinical studies, and one controlled trial. Given the great heterogeneity in the already reported studies, it was not possible to perform a formal metaanalysis. Therefore, experimental and clinical studies are discussed in the clinical context and their strengths as well as limitations are highlighted.

## Physiopathological arguments for vasopressor support in hemorrhagic shock

The early phase of hemorrhage is characterized by sympathetic system activation resulting in compensatory venous and arterial vasoconstriction, aimed at normalizing arterial blood pressure [4]. Initially, this mechanism is deemed efficacious. Animal experiments have even shown that when retransfusing the shed blood volume, previously withdrawn to produce hemorrhagic shock, arterial blood pressure values exceed those observed at baseline, underlining the performance and efficiency of compensatory mechanisms. Therefore, vasopressor support at this stage does not appear to be indicated [5]. However, several subsequent events are bound to progressively or brutally alter this vasoconstrictive vessel response. Beyond a certain amount of blood loss, sympathetic inhibition occurs, leading to a drop in vascular resistances and bradycardia, rapidly followed by cardiocirculatory arrest. During this phase immediately preceding cardiac arrest, and *a fortiori* in the case of cardiac arrest, the usefulness of rapid vasopressor injection to restore arterial blood pressure and redirect cardiac output toward vital organs is undisputable. Well before this phase of extreme exsanguination, animal models show that anesthetic agents exert a significant effect on the vasoconstrictive response to hemorrhage and that stable and normal blood pressure values seen in conscious animals are no longer observed in anesthetized animals following withdrawal of similar blood volumes [6]. In this context, it is important to remind that the majority of patients with hemorrhagic shock require the use of such agents to obtain analgesia or sedation for ventilatory intubation during shock management or before surgical intervention or embolization. Shock duration also is likely to modify the vasoconstrictive response. While in animal experiments involving short-duration hemorrhagic shock, the retransfusion of shed blood resulted in higher arterial blood pressure than baseline as previously mentioned, in long-duration shock exposure exceeding several hours, blood retransfusion alone does not allow arterial blood pressure to return to baseline values. This absence of normalization of blood pressure values suggests vasodilation, as seen in prolonged states of shock characterized by a deficiency of compensatory mechanisms [7]. In addition, an intense inflammatory response may develop in hemorrhagic shocks [8]. Blood withdrawal may cause global ischemia-reperfusion injuries resulting in up-regulation of cytokine expression [9] and oxidative and nitrosative stresses [10]. Thus, in a model of anesthetized rats, hemorrhagic shock led to a vascular hyporeactivity to norepinephrine mediated by an enhanced release of nitric oxide (NO), such as in septic shock [11]. Therefore, in such a context of insufficient vasoconstriction or even vasoplegia, vasopressor support seems justified. However, the difficulty when extrapolating animal data to humans must be stressed, as far as timing for vasopressor support is concerned.

In addition to their usefulness in the case of vasodilatation or insufficient vasoconstriction, vasopressors may be useful to restore hemodynamic parameters along with adequate vital organ infusion, thereby reducing the need for continuous fluid infusion, which may result in side-effects, such as tissue edema. The occurrence of a systemic inflammatory response, in particular an acute respiratory distress syndrome (ARDS), appears to be the primary cause of death on Day 3 in trauma patients [12]. In a series of 102 severely traumatized patients, the infusion of crystalloid solutions during the first 24 hours was associated with subsequent aggravation of pulmonary dysfunction [13]. Whilst in this study no direct causal relationship between fluid loading and pulmonary lesions could be established, limitation of sodium-water load during the initial resuscitation phase afforded by the vasoconstrictor use may be associated with beneficial effects during the secondary systemic inflammatory phase. It is probable that this is an entirely new indication for early vasopressor support. In addition, the benefits of a strategy based on vasopressor use and combining volume-sparing and optimized arterial blood pressure might have a positive impact on the progression of cerebral lesions (limitation of cerebral edema; maintenance of high infusion pressure), keeping in mind that cerebral lesions are eventually the first cause of death in trauma patients [12] and that even a single, brief episode of hypotension may worsen mortality [14]. Furthermore, in severely hypotensive patients with septic shock, Hamzaoui et al. showed that early administration of norepinephrine increased cardiac output, mediated by an increased cardiac preload likely due to venous constriction and by an increased cardiac contractility [15]. A similar effect may be expected in patients with hemorrhagic shock.

Lastly, in the case of abdominal lesions, early vasopressor infusion allowing portal output to decrease via splanchnic vasoconstriction (this effect being particularly pronounced following vasopressin use) is likely to result in decreased hemorrhagic loss from splanchnic blood vessels, while maintaining adequate infusion of other organs.

The question raised regarding vasopressor support for hemorrhagic shock is perhaps somewhat simplified, because it places all trauma and even all polytrauma patients under the same label of hemorrhagic shock. Shock in polytrauma patients is likely to be very different from single, hemorrhagic lesion. In these cases, the problem is not only hemorrhage, but also the multiple tissue injuries associated with prolonged shock, due to both hypovolemia as well as alveolar and tissue hypoxia, which subsequently trigger a hyperinflammatory response [16,17]. In this situation, vasopressor support at the very early stage appears particularly appropriate. To illustrate this, we refer to a clinical study in which vasoplegia and cardiac dysfunction were already noted in polytrauma patients at a very early stage, upon emergency room admission [16].

Although theoretically tempting, these concepts arguing for vasopressor support for hemorrhagic shock are only speculative, and animal and human studies must be further scrutinized to determine whether vasopressor agents are efficacious and at the same time devoid of detrimental ischemic side-effects.

## Experimental data

In animal models, when given at the early stage, vasopressors were shown to be capable of restoring a quasi-normal hemodynamic status by mobilizing nonconstraint venous blood volume and thus normalizing arterial blood pressure, cardiac output, and preload parameters, such as pulse pressure variations [18]. However, the consequences of such a masked hypovolemia are far from insignificant. In an experimental hemorrhage model associated with endotoxin injection, Hinder et al. investigated the effects of norfenefrine, a pure vasopressor [19]. These authors demonstrated that norfenefrine infusion, even in the absence of concomitant fluid loading, resulted in normalized filling pressures. This masked hypovolemia was associated with disseminated cardiac cell necrosis and severe acute renal dysfunction. This adverse effect is likely to be dose-dependent as reported by Poloujadoff et al. [20]. In this study, norepinephrine in combination with saline infusion in uncontrolled hemorrhagic shock in rats was associated with improved survival. But irrespective to the threshold of lower MAP (40 mm Hg versus 80 mmHg), the highest dose of norepinephrine was associated with increased mortality rate. These recent findings confirm those obtained in older studies, suggesting the deleterious effects of vasopressor use in experimental hemorrhage models, due to the worsening of tissue hypoperfusion [21]. Of note in all of these experimental models, the duration of shock was brief and vasopressor agents were used alone, most often without fluid challenge [18,19] at the early shock stage when arterial vasodilatation was not yet present. However, in a model of normovolemic hemorrhage in which pigs received hemodilution until death, maintaining MAP > 60 mmHg by norepinephrine infusion allowed for the exchange of a significantly higher volume of blood [22]. Using the pig model associating uncontrolled hemorrhage (splenic laceration) and cranial trauma, which is closer to the condition of a polytrauma patient, Alspaugh et al. have shown that early and isolated administration of phenylephrine was associated with a higher survival rate than with crystalloid fluid resuscitation alone, although the cardiac output was decreased in the phenylephrine group [23]. Of note, the splenic laceration resulted in large variability in hemorrhage volume and hemodynamic response of the individual animal. Using a similar model, Feinstein et al*.* compared crystalloid administration alone versus crystalloid + phenylephrine or vasopressin [24]. Vasopressor support limited the volume of perfused solutions, decreased pulmonary lesions, and reduced elevations of intracranial pressure. Recently, these observations have been confirmed using a similar model of cerebral lesions and hemorrhage, associating fluid infusion and vasopressin administration [25]. As the studies in head-injured patients showed that norepinephrine does not alter intracranial pressure [26,27], this protective effect of vasopressor infusion on intracranial pressure is likely to be dependent on the reduction of fluid loading rather than on a direct effect of the vasoconstrictive drug. Conversely in a model of uncontrolled hemorrhage induced by a penetrating liver trauma without brain injury, in comparison with hypertonic hydroxyethyl starch alone, the addition of norepinephrine did not show beneficial effect on cerebral perfusion pressure or brain tissue oxygenation [28]. These last studies underline the potential usefulness of vasopressor support in conditions where rapid blood pressure restoration is required (cranial trauma), as well as the effects of volume-sparing measures on pulmonary lesions and cerebral edema, and point out the need for initial fluid loading. Nonetheless, as a decrease in cardiac output and unfavorable metabolic effects (hyperlactatemia) were frequently observed in experimental studies associating fluid infusion and vasopressor support [29], the conditions for using a vasopressor agent along with fluid infusion must be better defined in terms of vasopressor agent and timing of infusion.

In experimental models of massive hemorrhage, several recent studies reported that vasopressin infusion was associated with survival benefits compared to fluid administration alone or to other vasopressor agents (epinephrine or norepinephrine) [30-33]. These models are characterized by a marked drop in arterial pressure before the initial resuscitation, arterial pressure being very close to central venous pressure (20 mmHg). In this context, the use of a vasopressor agent is primarily aimed at managing cardiac arrest, rather than treating hemorrhagic shock. Vasopressin may have a role to play as a lifesaving treatment in the case of hypovolemic cardiac arrest, designed to restore a satisfactory hemodynamic status before hemorrhage control. In addition, most experimental data come from the same group that focused their models on hepatic or other form of abdominal vascular injury resulting in a hemorrhage located in the splanchnic vascular bed [30,33,34]. It is thus possible to hypothesize that vasopressin, while significantly decreasing mesenteric flow and thus portal flow, might have the advantage of redistributing blood flow away from the hemorrhagic lesion in these models. However, this strategy may impair gut mucosal perfusion. Indeed, Stadlbauer et al. showed that infusion of vasopressin was followed by transient diarrhea during the reperfusion period, suggesting a potential gut ischemia. However, this side effect was transient and long-term survival was good [33]. The same team in a similar animal model also showed that vasopressin infusion did not result in significant cerebral injury as assessed with S100B protein measurement [34].

Despite the use of clinical relevant models of hemorrhagic shock (in particular by Poloujadoff et al. [20]), the experimental models are not able to reproduce exactly the conditions and multiple tissue lesions observed in real polytrauma patients. Moreover, most animal experiments only focused on the progression of shock states during a very short time period rarely exceeding 24 hours. Therefore, they do not reflect the complexity of a prolonged ICU stay. In summary, animal data suggest that unreasonable use of vasopressor agents at excessive doses and without fluid infusion appears to be detrimental. Yet, their use may be beneficial under better-controlled hemodynamic conditions. Because outside of these general settings, experimental animal data are considered incomplete, we cannot precisely define the conditions for the use of vasopressor agents.

## Clinical studies

To date, no clinical prospective study conducted in the hemorrhagic shock or traumatology setting compared different therapeutic strategies with and without vasopressor use. One, single, descriptive study reported on dopamine use in a general care protocol for polytrauma patients with life-threatening hemorrhage from pelvic fractures associating early arteriography ± embolization along with vasopressor treatment initiated within the first hour of hospital admission [1]. When comparing with historical series of similar patients, the outcome was better with aggressive therapeutic strategies. Because the vasopressor was administered in association with multiple other therapeutic bundles, and in absence of control group, the therapeutic effect of the vasopressor agent on patient outcome cannot be derived from this single study. Nevertheless, early use of vasopressor agents in hemorrhagic shock management was not associated with any obvious detrimental effects. Conversely, in a retrospective study conducted in the United States, early administration of any vasopressor was analyzed in a multivariable analysis. The authors showed that vasopressor use was associated with increased mortality, whereas aggressive crystalloid administration was found to be beneficial [35]. Of note, early deaths were excluded from analysis, although these very severe patients may be more likely to benefit from vasopressor infusion. More recently, Plurad et al. reported in a retrospective study in 1,349 selected patients without brain or spinal cord injuries that the early vasopressor use after the critical injury was associated with a significant increase in mortality rate regardless of the fluid status. Here too were only included the patients who had survived more than 24 hours [36]. Because the patients with early death may specifically benefit from early vasopressor use, the exclusion of these patients is a major limitation. These negative studies were recently challenged by a prospective study that assessed the effect of early vasopressin use in a double-blind, randomized trial. Compared with the control group (fluid alone, 40 patients), the addition of vasopressin (4 IU bolus followed by 2.4 IU/H for 5H, 38 patients) was associated with lower fluid resuscitation volume over 5 days (*p* = 0.04) with a mortality rate at day 5 of 25% versus 13%, respectively (*p* = 0.19) [37].

## Conclusions

Despite numerous theoretical arguments in favor of the relatively early vasopressor use in association with fluid infusion in hemorrhagic shock management, there is still insufficient clinical evidence to validate this strategy. Experimental data available to date clearly indicate that vasopressor support cannot be replaced by fluid loading. In contrast, when given in association with fluids, the use of vasopressor agents appears to offer advantages, at least in some experimental models. Nevertheless, the type of vasopressor and the precise timing still need to be defined. Lastly, no clinical studies have validated any vasopressor support for the management of hemorrhagic shock. The use of norepinephrine advocated by some teams appears reasonable, but it must be kept in mind that this recommendation is entirely based on expert opinions [38,39]. The North American strategy, which excludes vasopressors, may be explained by the wide differences in patients with hemorrhagic shock. As the European studies mainly recruited polytrauma patients, in which the inflammatory response to multiple tissue lesions begins early and lasts hours to days and often are associated with cerebral lesions that require achieving very strict arterial pressure goals, the recommendation of vasopressor use seems logical. In contrast, the North American recommendation of fluid loading alone may be related to a different epidemiologic pattern (frequent unique penetrating traumatic lesions). In these cases, the isolated lesion of a blood vessel is much closer to the experimental animal models involving “pure hemorrhagic shock,” which require less strict arterial pressure objectives, while awaiting prompt and final surgical hemostasis [40].

Following this discussion on vasopressor support, one should acknowledge that fluid loading is the first step to be considered in the management of hemorrhagic shock. Based on current information, further clinical studies that are designed to compare early vasopressor support plus fluid resuscitation versus fluid resuscitation alone are warranted. In this context, the prospective European study conducted to assess the impact of vasopressin infusion as a salvage therapy in prehospital hemorrhagic shock that persists despite standard treatment, including a first line vasopressor (Vasopressin In Traumatic Shock (VITRIS) trial, NCT00379522), may provide an answer.

## Competing interests

The authors declare that they have no competing interests.

## Authors’ contributions

All authors contributed to the drafting of the manuscript and approved the final version.
